# Bilateral bifid mandibular condyle associated with ankylosis of the temporomandibular joint in a 6-year-old child^☆^

**DOI:** 10.1016/j.radcr.2022.05.030

**Published:** 2022-06-22

**Authors:** Cihan Çelik, Abdinasir Mohamed Elmi, Faisal Abdi Osoble Osman

**Affiliations:** Radiology Department, Mogadishu Somali Turkey, Recep Tayyip Erdogan Training and Research Hospital

**Keywords:** The bifid mandibular condyle, Ankylosis, Bifid condyle, Temporomandibular joint, Computed tomography

## Abstract

Bifid mandibular condyle with ankylosis is an extremely rare condition and may arise as a developmental or traumatic defect. We report here a case of bilateral bifid mandibular condyle with ankylosis in a 6-year-old child. The patient had severe limitation of mouth opening and history of trauma 2 years ago.

## Introduction

The bifid mandibular condyle (BMC) is an extremely rare anatomic variation with a dubious etiology. It is a developmental disorder, but it has also been linked to trauma, infection, irradiation, vascular anomalies, abnormal muscle pull, condylar fractures, condylectomy, and nutritional, endocrine, genetic, or teratogenic factors [1]. The actual prevalence of BMC is debatable, ranging from 0.31% to 1.82% in previously published studies [2]. As a radiological finding, it is frequently asymptomatic. When symptomatic, it may be associated with pain, mouth opening limitation, ankylosis, facial asymmetry, and swelling in the affected region. For various diagnostic purposes, many types of temporomandibular joint (TMJ) imaging methods are used, including conventional radiography, computerized tomography (CT), MRI, ultrasonography, and cone beam CT scan [3]. Here, we report here a case of bilateral BMC with fibrous ankylosis in a 6-year-old child.

## Case study

**Fig. 1:**
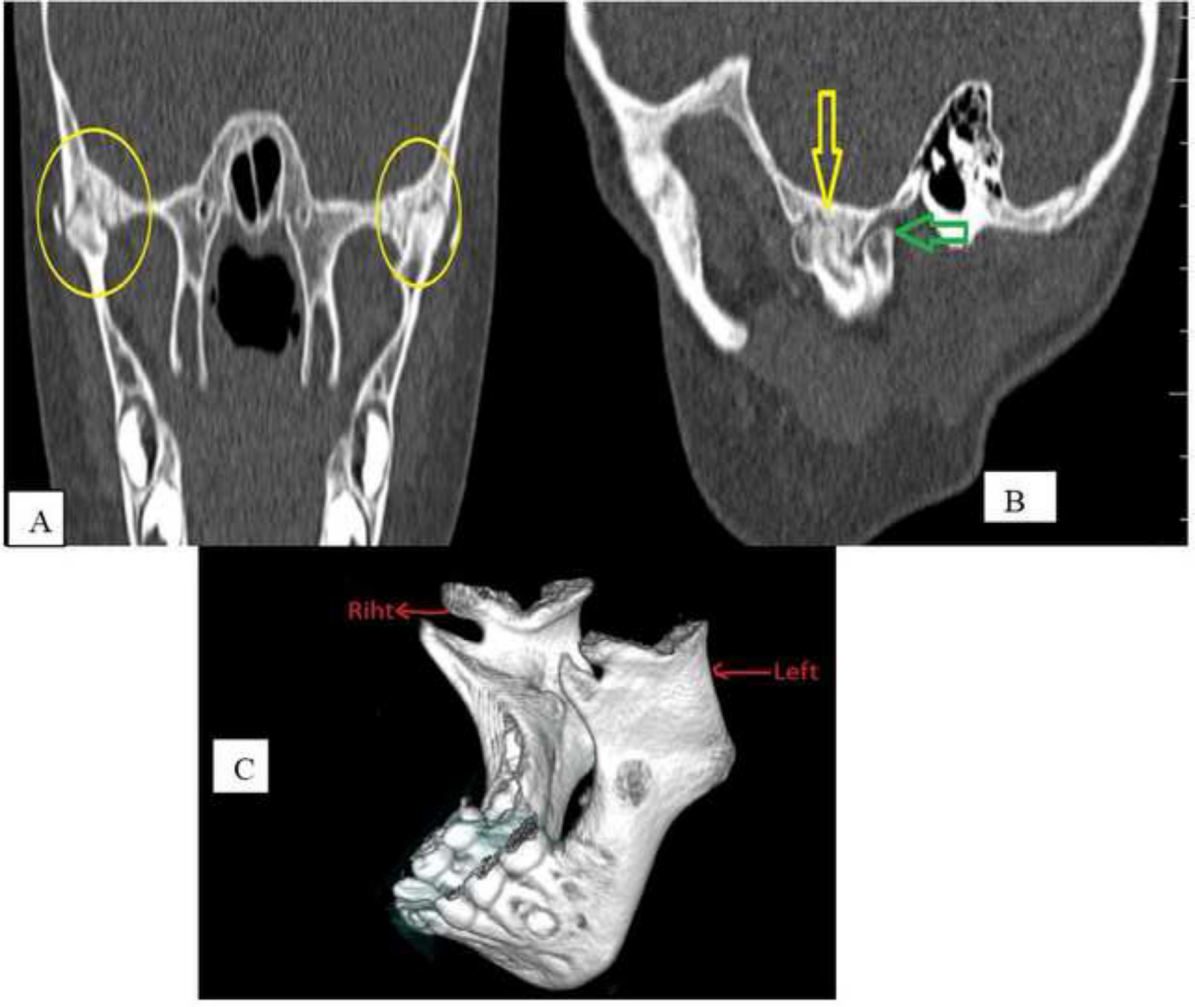
(A) Coronal reconstructed CT section of bilateral TMJ. Bilateral anterior head shows fibrous ankylosis with the temporal bone (yellow circles). (B) Sagittal reconstructed CT section through the lateral aspect of the right TMJ. The posteriorly placed condylar head is articulating with the temporal fossa (green arrow). The anterior head shows fibrous ankylosis with the temporal bone (yellow arrow). (C) Bilateral bifid mandibular condyle in 3-dimensional reconstructed image.

**Fig. 2:**
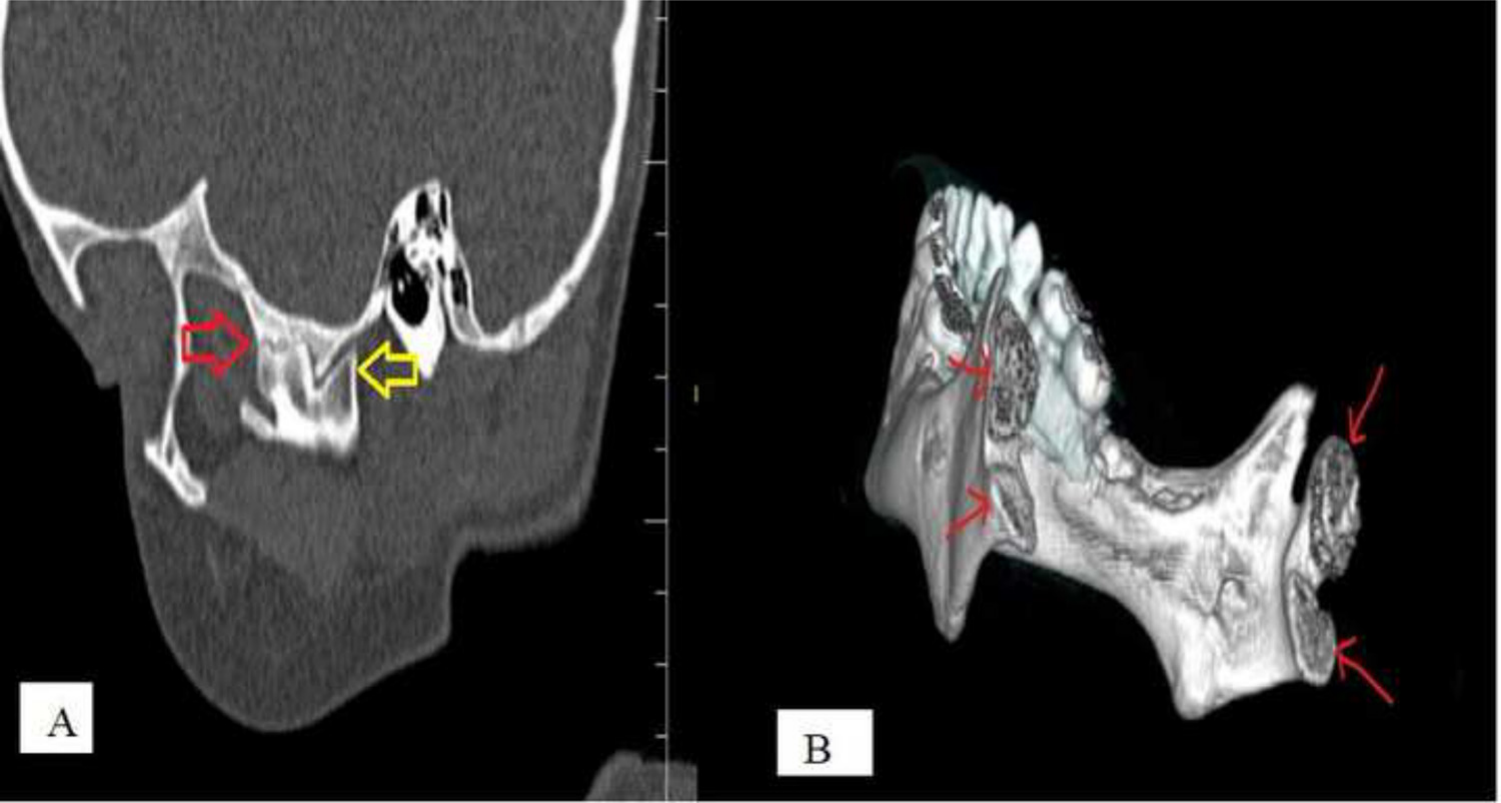
(A) Sagittal reconstructed CT section through the lateral aspect of the left TMJ. The posteriorly placed condylar head is flattened (yellow arrow). The anterior head shows fibrous ankylosis with the temporal bone (red arrow). (B) Three-dimensional reconstructed images show bilateral bifid mandibular condyle (BMC) formation (red arrows).

Somali Mogadishu R. T. E Research Hospital received a 6-year-old Somali child. His symptom was severe mouth opening limitation during speech and mastication. He had a trauma history 2 years ago falling from a tree. The family did not report any problems with reduced mouth opening and mastication before the trauma. His symptoms have worsened gradually since then. Upon initial physical examination, there was no facial asymmetry or bone fracture in the patient. We found the patient to have tenderness on palpation in his temporomandibular joints on both sides. A maximum jaw opening of 13 mm was achieved passively. CT scan showed a BMC associated with temporomandibular joint ankylosis. The anterior condyle has a false joint with the temporal bone, resulting in ankilosis and the posterior head articulating with the temporal fossa (Fig. 1). Both condyles were also displaced anteriorly. Fig. 2 shows the posterior condyle is flattened, and bilateral BMC in 3-dimensional reconstructed image.

## Discussion

The occurrence of a BMC is extremely rare. Hrdlicka attempted to explain the origin through the obstructed blood supply to the condyle during its development, resulting in the division to the condyle [4] when it was described as a condylar separation or groove of different depths in 1941.

Li et al. [5] described 4 fracture-related cases and classified their morphology based on the severity of the trauma, location, and relationship with the lateral pterygoid muscle. This muscle influences the direction of the fractured condylar piece and is an important factor in the formation of a BMC [2]. This theory is based on the fact that lateral pterygoid muscle activity causes an anteromedial displacement of the condyle after a condylar neck fracture. Then, through metaplasia, a new condylar head appears in the correct anatomic position, while the displaced condyle undergoes resorption [6,7].

Our patient has ankylosis of the temporomandibular joint and a trauma history from 2 years ago. Trauma is one of the most common causes of ankylosis. Rehman et al. [8] reported 10 cases of BMC in 37 patients with TMJ ankylosis in a retrospective study. Nine of the 10 cases were posttraumatic, while 1 was postinfectious [3]. In this case, the family reported trauma 2 years ago.

## Conclusion

We think that BMC disease associated with ankylosis affects the quality of life. And we assume that trauma is most likely the cause of the ankylosis.

## Patient consent

The patient was invited and written informed consent was obtained for his anonymized information to be published in this study.

## Ethics approval and consent to participate

Ethical approval for this study was waived by ethical committee of Mogadishu Somali Turkey, Recep Tayyip Erdogan Training and Research Hospital. The patient's parents were invited to participate and written informed consent was obtained**.**

## Authors' contributions

AME wrote the case report and discussion.

MK examined the radiological films and wrote the radiology report.

FAOO approval of the final version.

## Availability of data and materials

The data that support the findings of this study are available in Mogadishu Somali Turkey, Recep Tayyip Erdogan Training and Research Hospital information system. Data are however allowed to the authors upon reasonable request and with permission of the education and research committee.

## References

[1] Rajashri R., Periasamy S., Kumar S.P. (2021). Bifid mandibular condyle as the hidden cause for temporomandibular joint disorder. Cureus.

[2] Borrás-Ferreres J., Sánchez-Torres A., Gay-Escoda C. (2018). Bifid mandibular condyles: a systematic review. Med Oral Patol Oral Cirug Bucal.

[3] Miranda K., Carneiro A.S., Gerber J.T., Weiss S.G., Klüppel L.E., Scariot R. (2019). Treatment of atypical bifid mandibular condyle associated with ankylosis of the temporomandibular joint. Case Rep Surg.

[4] Hrdlička A. (1941). Lower jaw: double condyles. Am J Phys Anthropol.

[5] Li Z., Djae K.A., Li Z.B. (2011). Post-traumatic bifid condyle: the pathogenesis analysis. Dent Traumatol.

[6] López-López J., Ayuso-Montero R., Jané Salas E., Roselló-Llabrés X. (2010). Bifid condyle: review of the literature of the last 10 years and report of two cases. CRANIO®.

[7] Woo M.H., Yoon K.H., Park K.S., Park J.A. (2016). Post-traumatic bifid mandibular condyle: a case report and literature review. Imaging Sci Dent.

[8] Rehman T.A., Gibikote S., Ilango N., Thaj J., Sarawagi R., Gupta A. (2009). Bifid mandibular condyle with associated temporomandibular joint ankylosis: a computed tomography study of the patterns and morphological variations. Dentomaxillofac Radiol.

